# Presentation of Perforated Meckel’s Diverticulum and Phlegmon: A Case Report

**DOI:** 10.7759/cureus.53460

**Published:** 2024-02-02

**Authors:** Muhammad Muntazir Mehdi Khan, Asfia Arham, Rimsha Farooq, Simra Irfan, Rehman Alvi

**Affiliations:** 1 Surgery, The Ohio State University Wexner Medical Center, Columbus, USA; 2 General Surgery, Aga Khan University Hospital, Karachi, PAK

**Keywords:** vitelline duct remnant, gi tract, meckel´s diverticulum, surgical case reports, phlegmon, perforated meckel’s diverticulum

## Abstract

One of the most frequently encountered congenital anomalies of the gastrointestinal tract is the Meckel’s diverticulum. Perforation of the diverticulum, a rare complication, can significantly hinder accurate diagnosis of the condition. Other common complications associated with Meckel’s diverticulum include intestinal obstruction, intussusception, volvulus, inflammation, and hemorrhage. The presentation is similar to the presentation of appendicitis at times. Formation of a phlegmon around a perforated Meckel’s diverticulum can mask clinical signs and symptoms. We present a case of a 59-year-old man who presented with pain in the right upper and lower quadrants. After imaging, the patient underwent exploratory laparotomy, which revealed a perforated Meckel's diverticulum. This case highlights the importance of considering Meckel's diverticulum as a possible diagnosis in patients presenting with acute abdominal pain. A thorough approach to history and physical exam combined with imaging can help in the early diagnosis of a perforated Meckel’s diverticulum.

## Introduction

Meckel’s diverticulum is an uncommon occurrence in the adult population and may present in different ways [1]. It is considered the most common congenital malformation of the gastrointestinal tract [2-4]. Meckel’s diverticulum contains all layers of the gastrointestinal tract and is thus a true diverticulum [3]. It primarily contains ileal mucosa but might also contain mucosa from other parts of the gastrointestinal tract, including the stomach, pancreas, duodenum, and colon [3]. Heterotopic mucosa is present in 60% of the cases [2]. In patients presenting with acute abdominal pain, the presentation can often mimic acute appendicitis [5]. Even though the diagnosis is often confirmed on open laparotomy, diagnostic laparoscopy has also been a useful tool to diagnose and treat a perforated Meckel’s diverticulum [5]. It is rare for a Meckel’s diverticulum to present as bowel perforation. In this discussion, we present a patient diagnosed with a perforated Meckel’s diverticulum.

## Case presentation

**Figure 1:**
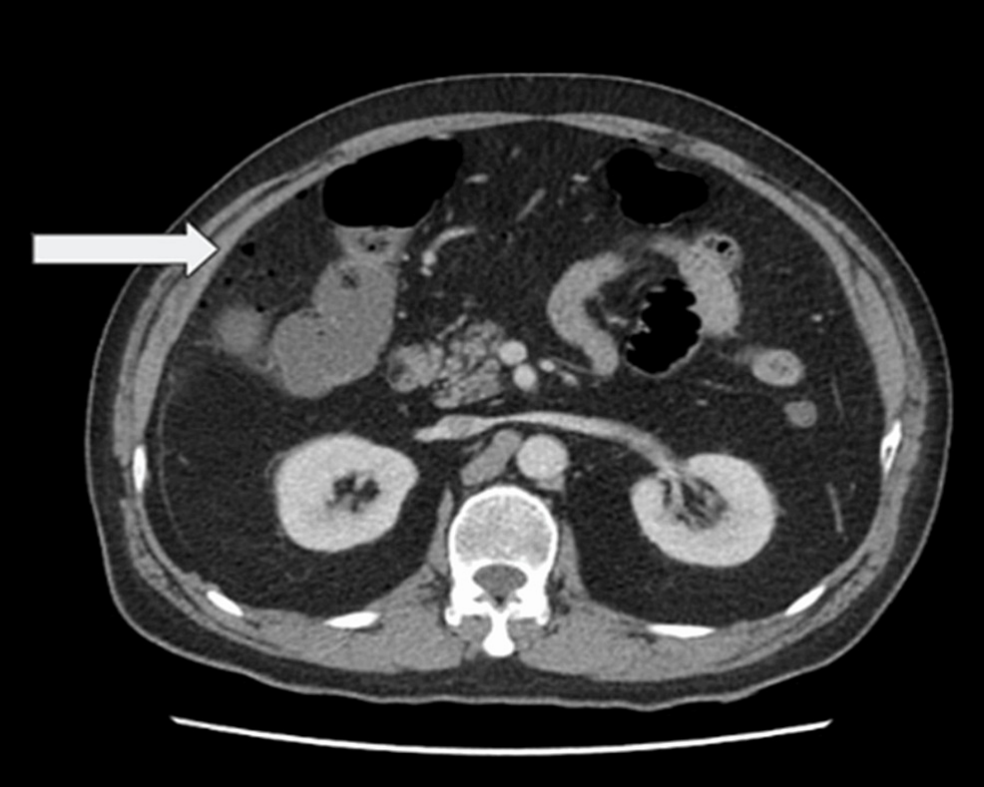
Abdominal CT scan showing free air specks in the abdominal cavity CT: Computerized tomography

**Figure 2:**
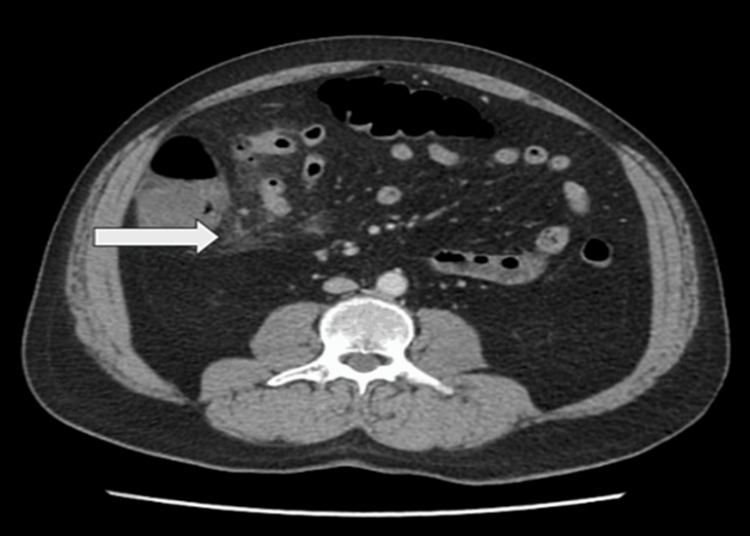
Abdominal CT scan showing fat stranding around the ileum CT: Computerized tomography

**Figure 3:**
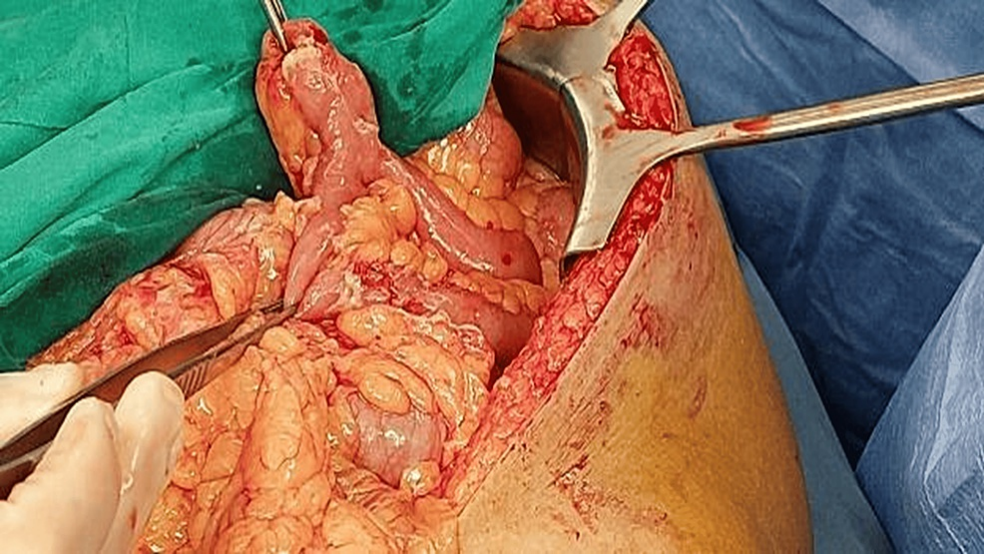
Intraoperative picture showing perforated Meckel’s diverticulum

A man in his late 50s, with no known comorbidities, presented to the emergency room with the primary complaint of severe abdominal pain that started 15 days back. Initially, the patient had diffuse pain in the periumbilical area that had now migrated to the right upper and right lower quadrants and was progressively worsening. There was no history of recurrent abdominal pain in the past before this episode. The patient also reported experiencing nausea for the past 24 hours. The patient denied experiencing diarrhea and constipation or noticing fresh or old blood in his stool. It was noted that the patient had undergone an appendectomy almost 40 years ago. Upon examination, the patient’s vital signs were stable and a physical examination revealed abdominal distension, significant tenderness in the right upper and right lower quadrants, and guarding in the right side of the abdomen. Rovsing's sign, obturator, and psoas signs were all negative. There was no rebound tenderness, bowel sounds were decreased, and digital rectal examination was normal. Laboratory investigations showed a hemoglobin of 13.2, a white blood cell count of 13.6 with 88.1% neutrophils. Electrolytes, pancreatic enzymes, and renal parameters were within normal range. The patient was admitted to the hospital, was given broad-spectrum antibiotics, and underwent a computerized tomography (CT) scan. The CT scan revealed flecks of extra-luminal air, indicating pneumoperitoneum (Figure 1), and peri-ileal fat stranding (Figure 2). Subsequently, owing to the patient's deteriorating condition, the patient underwent an exploratory laparotomy, during which a perforated Meckel’s diverticulum with phlegmon was observed (Figure 3). The Meckel’s diverticulum was excised from the base using a linear stapler, and pus was found in the sub-hepatic space. An abdominal washout was performed using normal saline. The patient developed postoperative ileus, but he was managed by nasogastric tube decompression. The patient was discharged in a stable condition on the fifth day after surgery. The histopathology report identified a Meckel’s diverticulum without the presence of heterotopic mucosa.

## Discussion

Meckel’s diverticulum is a well-documented congenital malformation of the gastrointestinal tract [2,5]. It is present in approximately 2% of the general population [2,5]. Intestinal obstruction (41%), intussusception (17%), volvulus (5.5%), inflammation (13%), and hemorrhage (34%) are the most common complications associated with Meckel’s diverticulum [2,6]. It is rare for a Meckel’s diverticulum to present as perforation, only present in 0.1-21% of symptomatic patients with Meckel's diverticula. [3,6]. It is usually due to a stercolith causing obstruction of the diverticulum and then inflammation and necrosis [3,6]. It can result in perforation and present as such, making the diagnosis confusing [6].

Diagnosis of a perforated Meckel’s diverticulum is not easy especially if CT is not available [7]. Because of its presentation mimicking that of appendicitis, it is important for doctors to keep in mind the presentation of a locally cordoned-off perforated Meckel’s diverticulum due to surrounding phlegmon if appendicitis has been ruled out through history, physical examination, and radiological findings. This is especially important for patients who have undergone a prior appendectomy [8]. Other possible causes of right-sided abdominal pain, which include diverticulitis, gallstone disease, hepatitis, inflammatory bowel disease, and renal colic, should be kept in mind during the evaluation of such patients. A thorough approach to history and physical exam combined with imaging can aid in early diagnosis of a perforated Meckel’s diverticulum [8].

Laparotomy or laparoscopic approaches are simultaneously diagnostic and therapeutic [3,5]. In uncomplicated acute diverticulitis, nonoperative management including bowel rest and antibiotics is preferred over operative management. Double balloon enteroscopy is a new and advanced tool that can aid in the diagnosis and further direct treatment options [9,10].

## Conclusions

The presentation of Meckel’s diverticulum is often like the presentation of acute appendicitis. Therefore, in patients with a history of appendectomy presenting with right-sided abdominal pain, Meckel’s diverticulitis should always be considered as a possible diagnosis. The presence of phlegmon around the ruptured Meckel’s diverticulum can sometimes mask the signs and symptoms of peritonitis, leading to delayed diagnosis and treatment. Early intervention through diagnostic and therapeutic laparotomy or laparoscopy is crucial to improve the management and overall outcome of the patient.
